# A Second-Order Network Structure Based on Gradient-Enhanced Physics-Informed Neural Networks for Solving Parabolic Partial Differential Equations

**DOI:** 10.3390/e25040674

**Published:** 2023-04-18

**Authors:** Kuo Sun, Xinlong Feng

**Affiliations:** College of Mathematics and System Sciences, Xinjiang University, Urumqi 830046, China; sunkuo@stu.xju.edu.cn

**Keywords:** physics-informed neural networks, second-order neural network, deep mixed residual method, parabolic partial differential equations

## Abstract

Physics-informed neural networks (PINNs) are effective for solving partial differential equations (PDEs). This method of embedding partial differential equations and their initial boundary conditions into the loss functions of neural networks has successfully solved forward and inverse PDE problems. In this study, we considered a parametric light wave equation, discretized it using the central difference, and, through this difference scheme, constructed a new neural network structure named the second-order neural network structure. Additionally, we used the adaptive activation function strategy and gradient-enhanced strategy to improve the performance of the neural network and used the deep mixed residual method (MIM) to reduce the high computational cost caused by the enhanced gradient. At the end of this paper, we give some numerical examples of nonlinear parabolic partial differential equations to verify the effectiveness of the method.

## 1. Introduction

With the development of computational technology, neural network theory, and data science, deep learning algorithms have been remarkably successful in various application areas [1,2,3]. In physics and engineering, many important physical models are described as partial differential equations (PDEs), such as Navier–Stokes equations [4] for fluid mechanics and Maxwell equations [5] for electromagnetic field theory. When solving partial differential equations using traditional numerical methods, such as the Finite Difference Method (FDM) [6], Finite Element Method (FEM) [7], Finite Volume Method (FVM) [8], Radial Basis Function Method (RBF) [9], etc., problems such as high computational costs and the curse of dimensionality are often encountered. Over the last few years, the use of machine learning to solve partial differential equations has also rapidly expanded [10,11,12,13]. In 2018, Karniadakis and his research team were the first to put forward the concept of physics-informed neural networks (PINNs) [14,15], which solve PDEs by embedding them and their initial boundary value conditions in the loss function of the neural network thanks to automatic differentiation [16], following the physical laws described by nonlinear partial differential equations. Compared to traditional methods, PINNs are independent of the grid’s quality, can avoid the curse of dimensionality [17], and offer significant advantages in solving forward and inverse problems for partial differential equations. They also have great advantages in high-dimensional problems and complex geometric region problems. They have been extensively applied in various domains involving partial differential equations [18,19,20,21,22].

Although PINNs have achieved good results in solving partial differential equations, there are still some aspects that need improvement, such as their accuracy and efficiency when used to solve complex problems. Several approaches have been proposed to improve PINNs’ performance, for example, residual-based adaptive refinement (RAR) [23], importance sampling [24], adaptive activation function [25,26,27], adaptive weight [28,29,30], etc. These strategies can go a long way toward improving the neural network’s performance. Meng et al. proposed the parareal physics-informed neural network (PPINN) [31], which decomposes a long-time problem into multiple short-time problems for parallel iterative solutions, which can accelerate the training speed of PINNs. Additionally, a PINN algorithm based on domain decomposition was also proposed [32,33,34,35], as well as other extension algorithms [36,37,38]. Likewise, the theoretical analysis of PINNs presents numerous difficulties. Nevertheless, Mishra et al. derived a generalization error estimate according to the training error and the number of training samples by taking advantage of the stability characteristics of partial differential equations [39]. During this time, in [40], for a particular type of inverse problem, it was also proven that PINNs produce a strict estimation of the generalization error when approximating these inverse problems. Ryck et al. [41] placed a strict upper bound on the errors produced by PINNs and extended physics-informed neural networks (xPINNs) when solving Navier–Stokes equations under certain preconditions.

It is well known that increasing the width and depth of a network can improve network performance, and deep networks are generally better than shallow ones, but they are harder to train. A residual neural network [42,43] with a jump connection structure can be very effective in avoiding gradient vanishing [44] and can train deeper networks. Based on a residual network, Ruthotto et al. [45] established a connection between a deep residual convolutional neural network and a partial differential equation and constructed a second-order convolutional neural network for image recognition through a second-order hyperbolic equation. With this idea in mind, here, we constructed a second-order network structure based on a parametric light wave equation. In order to improve the performance of the neural network, we also used an adaptive activation function strategy and a gradient-enhanced strategy [46] and combined the deep mixed residual method (MIM) [47] to reduce the calculation cost of high-order derivatives. We applied this method to solve forward and inverse problems for partial differential equations. The heat conduction and diffusion of a substance appear to be processes of entropy change, which can be described by parabolic partial differential equations [48,49]. This is also a motivation for this paper to explore the deep learning algorithm for solving parabolic partial differential equations.

The rest of this document is arranged in the following manner: In Section 2, we take a brief look at solving differential equations through physics-informed neural networks. In Section 3, the methodology proposed in this paper is presented in detail. In Section 4, several numerical examples are given to test the efficiency of the proposed method in the resolution of differential equations, including forward and inverse problems. Finally, in Section 5, we summarize this work.

## 2. PINNs for Solving PDEs

This section introduces physics-informed neural networks (PINNs) for solving partial differential equations. Mathematically, a deep neural network can be regarded as a composite function of the input variable x, with a very high capacity for approximation. A neural network with a depth of *L* has only 1 input layer, L−1 hidden layers, and 1 output layer. Simultaneously, we assume that there are $N_{k}$ neurons in the *k* layer, so the corresponding weight matrix and bias vectors are $W^{k}\in R^{N_{k}\times N_{k-1}}$ and $b^{k}\in R^{N_{k}}$, respectively. Then, we take into account a nonlinear activation function $f_{act}$, including logistic sigmoid ($1/\left(1+e^{-x}\right)$), hyperbolic tangent (tanh) ($\left(e^{x}-e^{-x}\right)/\left(e^{x}+e^{-x}\right)$), the rectified linear unit (ReLU) ($\max\left\{0,x\right\}$), etc. Finally, the neural network can be recursively expressed in the following way [25]:

(1) $$y\left(x,\Theta \right)=W^{L}\left(f_{act}\left(W^{L-1}\left(\cdots f_{act}\left(W^{2}\left(f_{act}\left(W^{1}x+b^{1}\right)\right)+b^{2}\right)\cdots \right)+b^{L-1}\right)\right)+b^{L},$$

where $\Theta ={\left\{W^{i},b^{i}\right\}}_{i=1}^{L}$ denotes the trainable parameters in the neural network. Additionally, we consider the following spatiotemporal PDEs for the solution $u\left(x,t\right)$ parameterized by the parameter λ defined on the domain $\Omega \times \left(0,T\right)$ [46]:

(2) $$\left\{\begin{array}{cc} F(x,t,u,u_{t},\nabla u,\Delta u,\lambda ) & =0,\left(x,t\right)\in \Omega \times \left(0,T\right),\\ Bu & =g,\left(x,t\right)\in \partial \Omega \times \left(0,T\right),\\ u & =h,\left(x,t\right)\in \Omega \times \left\{0\right\}, \end{array}\right.$$

where $\left(x\right)=\left(x_{1},x_{2},\cdots ,x_{d}\right)\in R^{d}$, λ is a constant or can be a function of x and *t*, $\Omega \subset R^{d}\left(d\in N^{+}\right)$ is the *d*-dimensional physical domain, *T* is the upper limit of the time interval, and B is a representation of the boundary conditions, including the Dirichlet boundary condition, Robin boundary condition, etc.

**Figure 1:**
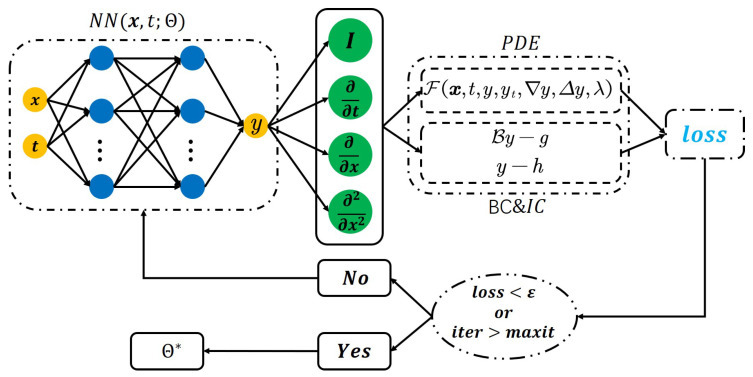
Schematic of PINNs for Equation (2).

Physics-informed neural networks are a method in which a partial differential equation and its initial boundary value conditions are added to the loss function using automatic differentiation (AD) [16]. Our objective is to find the optimal weights for which the correctly defined loss function is minimized. For Equation (2), we typically use the Mean Square Error (MSE) to construct the loss function, which is formed as follows [50]:

(3) $$\mathrm{Loss}=L_{f}+L_{b}+L_{i},$$

where

$$\begin{array}{cc} L_{f} & =\frac{1}{\left|T_{f}\right|}\sum_{\left(x,t\right)\in T_{f}}{\left|F(x,t,y,y_{t},\nabla y,\Delta y,\lambda )\right|}^{2},\\ L_{b} & =\frac{1}{\left|T_{b}\right|}\sum_{\left(x,t\right)\in T_{b}}{\left|By-g\right|}^{2},\\ L_{i} & =\frac{1}{\left|T_{i}\right|}\sum_{\left(x,t\right)\in T_{i}}{\left|y-h\right|}^{2}, \end{array}$$

where $T_{f}$, $T_{b}$, and $T_{i}$ represent the set of PDE residual training points, the set of boundary training points, and the set of initial training points, respectively. The PINN framework [23] can be found in Figure 1. In some PDEs, boundary conditions can be enforced accurately and automatically by modifying the network architecture [51,52,53,54], eliminating the term associated with the boundary conditions from the loss function (3).

## 3. Methodology

**Figure 2:**
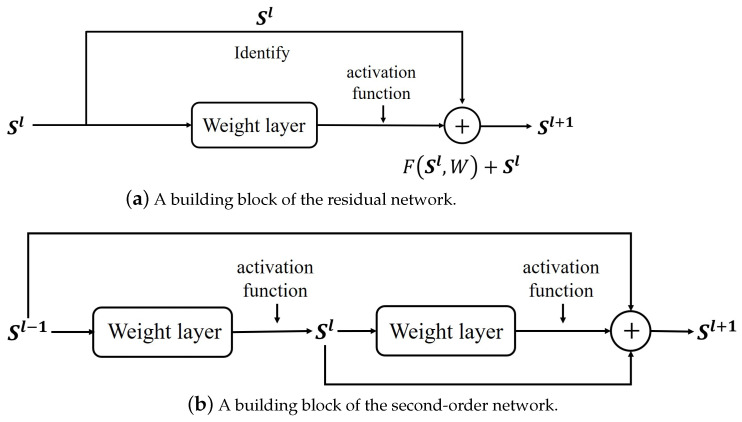
A building block.

When PINNs are used to solve differential equations, sigmoid and tanh are typically used as nonlinear activation functions that take part in neural network training. If the input dataset is very small or very large, the sigmoid and tanh derivatives will be around 0, and the gradient update will slow down during backpropagation [55]; the vanishing gradient problem [44] will inevitably occur. He et al. present a residual learning framework [42,43] to train deeper networks and avoid gradient vanishing. This neural network framework adopts residual machine learning every few hidden layers, and a building block is shown in Figure 2a [42]. The building block is defined as [42]:

(4) $$S^{l+1}=F\left(S^{l},W\right)+S^{l}.$$

Here, $S^{l}$ and $S^{l+1}$ are the input and output vectors of the *l*-layers considered, and the function $F\left(S^{l},W\right)$ represents the residual mapping to be learned. The example in Figure 2a has one layer and $F\left(S^{l},W\right)=f_{act}\left(W^{l+1}S^{l}+b^{l+1}\right)$.

The residual network in Equation (4) can be seen as the forward Euler formula [45,56]:

(5) $${\left.\frac{\partial S}{\partial t}\right|}_{\left[l,\;l+1\right]}\approx \frac{S^{l+1}-S^{l}}{\delta_{t}}=F\left(S^{l},W\right).$$

Therefore, the residual network can be represented by a first-order differential equation.

Similarly, different network structures can be constructed by considering different numerical schemes for hyperbolic PDEs. For a second-order dynamical system of the form [45]:

(6) $$\frac{\partial^{2}S}{\partial t^{2}}=F\left(S,W\right),$$

Ruthotto et al. established the relationship between a deep residual network and partial differential equations. They obtained a second-order convolutional neural network in the following form by discretizing the above second-order equation [45]:

(7) $$S^{l+1}=2S^{l}-S^{l-1}+\delta_{t}^{2}F\left(S^{l},W\right).$$

The building block of the second-order network is shown in Figure 2b. In this study, the structure of the proposed second-order neural network was constructed using a parametric light wave equation in the following form [57]:

(8) $$\frac{\partial S}{\partial t}+\varepsilon \frac{\partial^{2}S}{\partial t^{2}}=F\left(S,W\right).$$

We can obtain this by discretizing Equation (8) using the central difference:

(9) $$\left(\frac{1}{2\delta_{t}}+\frac{\varepsilon }{\delta_{t}^{2}}\right)S^{l+1}=F\left(S^{l},W\right)+\frac{2\varepsilon }{\delta_{t}^{2}}S^{l}+\left(\frac{1}{2\delta_{t}}-\frac{\varepsilon }{\delta_{t}^{2}}\right)S^{l-1}.$$

It can be seen that Formulas (7) and (9) have the same form, except that the coefficients of each term are different. Therefore, the network represented by Formula (9) is still a second-order network.

We used a network structure like that in Formula (9) for PINNs. For this network structure, we used a simple linear combination and nonlinear mapping of the activation function for the first hidden layer, the residual connection shown in Figure 2a for the second hidden layer, and the connection mode given by Equation (9) and Figure 2b for other hidden layers. Finally, we added a linear output layer. The feedforward neural network can be recursively expressed as follows:

(10) $$\begin{array}{ccc} \mathrm{input}:\; & S^{0}=x; & \\ \mathrm{hidden}\;\mathrm{layer}\;1:\; & S^{1}=f_{act}\left(W^{1}S^{0}+b^{1}\right); & \\ \mathrm{hidden}\;\mathrm{layer}\;2:\; & S^{2}=\delta_{t}f_{act}\left(W^{2}S^{1}+b^{2}\right)+S^{1}; & \\ \mathrm{hidden}\;\mathrm{layer}\;l:\; & S^{l}=\frac{2\delta_{t}^{2}}{\delta_{t}+2\varepsilon }f_{act}\left(W^{l}S^{l-1}+b^{l}\right)+\frac{4\varepsilon }{\delta_{t}+2\varepsilon }S^{l-1}+\frac{\delta_{t}-2\varepsilon }{\delta_{t}+2\varepsilon }S^{l-2},\mathrm{for}\;\;3\leq l\leq L-1; & \\ \mathrm{output}:\; & S^{L}=W^{L}S^{L-1}+b^{L}. \end{array}$$

Here, $S^{L}=y\left(x,\Theta \right)$ is the neural network’s output. It can be seen from the above expression that, compared with Equation (1), when the neural network has the same depth and width, the neural network expressed in Equation (10) has a stronger degree of nonlinearity, which can be said to be more expressive. This is a kind of neural network structure based on the difference scheme, and this is also a feasible scheme if pursuing structural diversity in neural networks. Ruthotto et al. [45] proved that the forward propagation of a second-order network is stable under certain conditions, which provides a foundation for our subsequent theoretical research. We hope that the continuous deep neural network (DNN) model can lead to some new breakthroughs in theory and algorithms, help simplify network architecture design, reduce trial and error, and improve training accuracy.

The adaptive activation function strategy [25,26,27] accelerates convergence and improves accuracy. We utilized Neuron-wise locally adaptive activation functions (N-LAAFs) [26] to enhance the performance of the neural network. It is assumed that $x^{ij}$ is the input of the *j*-th neuron of the *i*-th hidden layer, $y^{ij}$ is the output of this neuron, and $a^{ij}$ is a hyperparameter that is added to this neuron in the following format [26]:

(11) $$y^{ij}=f_{act}\left(na^{ij}x^{ij}\right),i=1,2,\cdots ,L-1,j=1,2,\cdots ,N_{j}.$$

In this case, $n\in N^{+}$ is a predetermined scale factor.

When the PDE residual equals zero, the corresponding gradient must equal zero. The gradient-enhanced PINNs (gPINNs) proposed in [46] force the derivatives of the PDE residual to be zero as well, i.e.,

(12) $$\nabla F\left(x,t\right)=\left(\frac{\partial F}{\partial x_{1}},\cdots ,\frac{\partial F}{\partial x_{d}},\frac{\partial F}{\partial t}\right)=0,\left(x,t\right)\in \Omega \times \left(0,T\right).$$

This will add an additional loss term [46] to the loss function (3):

(13) $$L_{g}=\sum_{k=1}^{d+1}\left(\frac{1}{\left|T_{g_{k}}\right|}\sum_{\left(x,t\right)\in T_{g_{k}}}|\nabla_{k}F{|}^{2}\right).$$

Here, $\nabla_{k}F$ is the *k*-th element of ∇F(x,t), and $T_{g_{k}}$ is the set of residual points for the derivative $\nabla_{k}F$. In this paper, let us set $T_{g_{1}}=\cdots =T_{g_{d+1}}=T_{g}$, $T_{g}$, and $T_{f}$ as the same dataset.

In order to reduce the high computational cost caused by gPINNs, we used the deep mixed residual method (MIM) [47]. We illustrate this with the heat equation, where F in Equation (2) is $F:=u_{t}-u_{x_{1}x_{1}}-u_{x_{2}x_{2}}-f=0$. Then, we introduce the auxiliary variables *p* and *q*, and let $p:=u_{x_{1}}$ and $q:=u_{x_{2}}$; then, the PDEs’ governing equations are rewritten as follows:

(14) $$\left\{\begin{array}{cc} \; & p=u_{x_{1}},q=u_{x_{2}}\\ \; & u_{t}-p_{x_{1}}-q_{x_{2}}=f \end{array}\right..$$

Here, the auxiliary variables *p* and *q* are the outputs of the feedforward neural network. In this paper, we use MIM and make $v=F(x,t,y,y_{t},\nabla y,\Delta y,\lambda )$, and then the loss term $L_{g}$ is rewritten as:

(15) $$L_{g}=\frac{1}{\left|T_{g}\right|}\sum_{\left(x,t\right)\in T_{g}}{\left|F-v\right|}^{2}+\frac{1}{\left|T_{g}\right|}\sum_{k=1}^{d+1}(\sum_{\left(x,t\right)\in T_{g}}|\nabla_{k}v{|}^{2}).$$

Finally, we rewrite Formula (3) as follows:

(16) $$\mathrm{Loss}=L_{f}+L_{b}+L_{i}+L_{g}.$$

**Figure 3:**
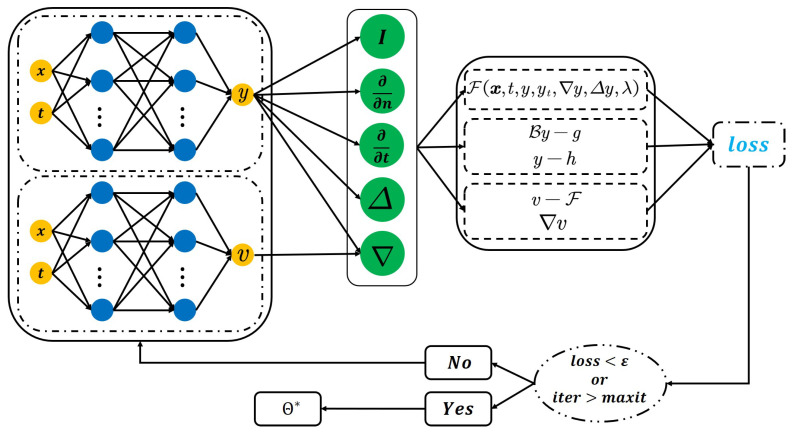
Schematic of gPINNs based on MIM for the Equation (2).

The algorithm framework can be found in Figure 3 and Algorithm 1. In this study, for neural networks $y(x,\Theta_{1})$ and $v(x,\Theta_{2})$, the same structural parameters are used unless otherwise specified. We used L-BFGS [58] as the optimization algorithm of the neural network to minimize the loss function (16) in the neural network training process. The initialization method for trainable parameters’ weights is the Xavier method [59], and the biases are initialized to zero. All randomly sampled point locations were generated using a Latin Hypercube Sampling strategy [60]. Meanwhile, the initial parameter selection for the hyperparameters *n* and $a^{ij}$ are chosen such that $na^{ij}=1$ [25,26,27].

Algorithm 1 shows the specific steps of the presented method compared with the existing PINN algorithm [14,26,46,47]. The main difference is that in the second and third steps, using two neural networks, a neural network is used to approximate the solutions of differential equations, and another is used to approximate the PDEs’ governing equations.
**Algorithm 1** gPINN algorithm based on MIM.Construct the training dataset $T_{f}$, $T_{b}$, $T_{i}$, and $T_{g}$ for the PDE residual, boundary conditions, initial conditions, and PDE residual gradient.Construct two neural networks $y(x,\Theta_{1})$ and $v(x,\Theta_{2})$ with the parameter $\Theta =\left\{\Theta_{1},\Theta_{2}\right\}$ using (10), where $\Theta_{k}={\left\{W_{k}^{i},b_{k}^{i}\right\}}_{i=1}^{L}\cup {\left\{a_{k}^{ij}\right\}}_{i=1:L-1}^{j=1:N_{j}},\left(k=1,2\right)$.Construct the PDE residual F and the PDE residual gradient $\tilde{\nabla }F$ using neural networks $y(x,\Theta_{1})$ and $v(x,\Theta_{2})$ in the governing equations using automatic differentiation.Construct the loss function using the Mean Square Error.Train the neural networks to find the best parameters $\Theta^{*}$ to minimize the loss function.

## 4. Numerical Examples

In this section, we give several examples to check the efficiency of the above method in the resolution of partial differential equations. On the test set, we used the Rel.$L^{2}$ error to evaluate the performance of neural network training [61]:

(17) $$Rel.L^{2}\mathrm{error}=\frac{\sqrt{\sum_{i=1}^{N}{\left|y^{*}\left(x^{i},t^{i}\right)-u\left(x^{i},t^{i}\right)\right|}^{2}}}{\sqrt{\sum_{i=1}^{N}{\left|u\left(x^{i},t^{i}\right)\right|}^{2}}},$$

where $y^{*}$ represents an optimal approximative solution derived from neural network training, *i* represents the test point number, and *N* represents the number of test points. In this part of the forward and inverse problem examples, the default values of ε and $\delta_{t}$ in Equation (10) are both set to 1.

### 4.1. Forward Problem

#### 4.1.1. Burgers’ Equation

Burgers’ equation is a nonlinear partial differential equation that simulates the propagation and reflection of a shock wave. It is used in fluid mechanics, nonlinear acoustics, gas dynamics, etc. First, we chose the 2D viscous Burgers’ equation as the first example in this document, which takes the following form [62]:

(18) $$\left\{\begin{array}{cc} u_{t}+uu_{x_{1}}+uu_{x_{2}} & =\nu \left(u_{x_{1}x_{1}}+u_{x_{2}x_{2}}\right),\;\left(x_{1},x_{2},t\right)\in {\left(-10,10\right)}^{2}\times \left(0,10\right),\\ u & =g,\;\left(x_{1},x_{2},t\right)\in \partial {\left(-10,10\right)}^{2}\times \left(0,10\right),\\ u & =h,\;\left(x_{1},x_{2},t\right)\in {\left(-10,10\right)}^{2}\times \left\{0\right\}, \end{array}\right.$$

where the parameter ν is the diffusion coefficient, the viscosity of the material, and we set the parameter ν=0.1. The initial and boundary conditions are derived from the exact solution: $u\left(x_{1},x_{2},t\right)=\frac{1}{1+\exp\left(\left(x_{1}+x_{2}-t\right)/2\nu \right)}$. Here, we aim to infer the entire spatiotemporal solution of the above Equation (18) for this example. For the neural network structure, we used five hidden layers, each with 50 neurons; the scale factor *n* is 50, and the tanh function is the activation function for this example. Meanwhile, for training, we constructed a training set of 3,000 boundary training points, 500 initial training points, and 10,000 PDE residual training points. We used the above configuration to form a neural network to obtain an approximate solution that satisfies Equation (18).

**Figure 4:**
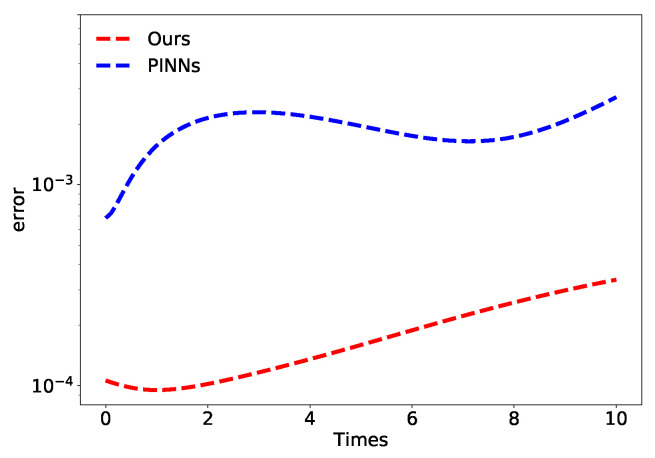
Burgers’ equation: Rel.$L^{2}$ error over time.

**Figure 5:**
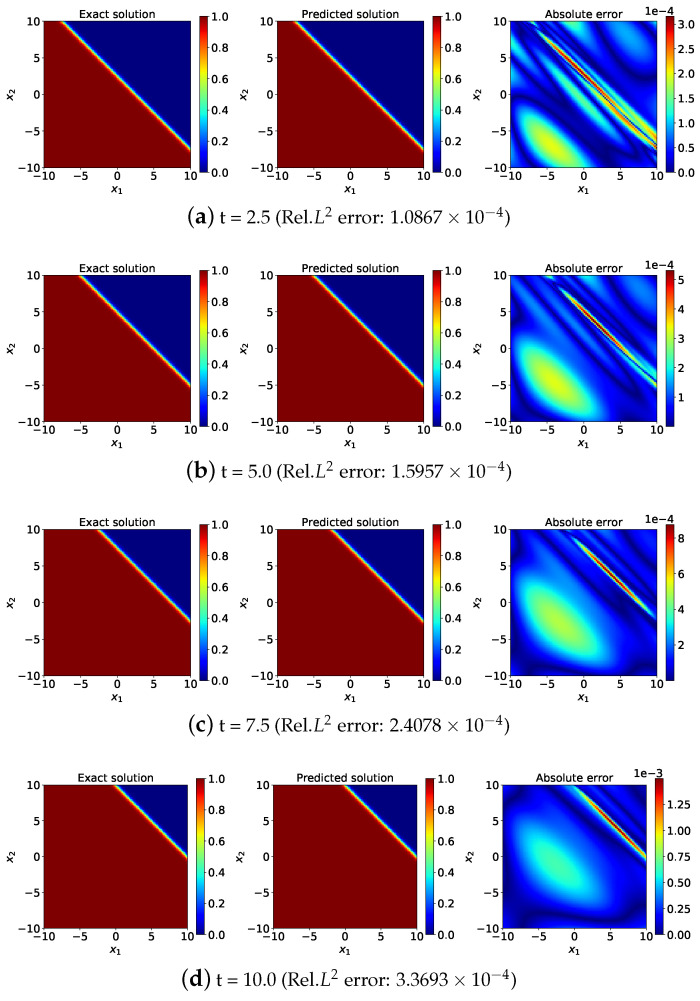
Burgers’ equation: exact solution, predicted solution, and absolute pointwise error at different times.

Next, we show the results of training. The Rel.$L^{2}$ error curve of Equation (18) on the test set over time is shown in Figure 4. It can be seen that the accuracy of the proposed method is about 7∼8 times higher than that of PINNs. Meanwhile, the exact solution, predicted solution, and error distributions at four time points, t=2.5, t=5.0, t=7.5, and t=10.0, are plotted in Figure 5. It can be seen in the error distribution diagram that the areas with large errors are mainly concentrated near the discontinuous point of the function, which can be improved by using a residual-based adaptive refinement (RAR) method [23]. This will be a future research direction for discontinuous solutions and large gradient changes. Clearly, the proposed method provides a good approximation of the equation. We present a comparative analysis in the Appendix A, including the benchmark PINNs method, adaptive activation function, gPINNs method, and the method mentioned in Section 3 of this paper.

#### 4.1.2. Taylor–Green Flow

When we consider incompressible Navier–Stokes equations (N-S equations), the governing equations can be written in a dimensionless form as [4]:

(19) $$\left\{\begin{array}{cc} u_{t}+\left(u\cdot \nabla \right)u & =-\nabla p+\nu \Delta u+f,\\ \nabla \cdot u & =0, \end{array}\right.$$

where $u=(u_{1},u_{2})$ is a velocity vector field, *p* denotes the fluid pressure, f is a given source term, ν=1/Re denotes the kinematic viscosity of the fluid, and Re is the Reynolds number.

We proceeded to model a time-dependent flow problem, i.e., the Taylor–Green flow, using the proposed method. The computational domain is defined as $\left(x_{1},x_{2},t\right)\in {\left(-\pi ,\pi \right)}^{2}\times \left(0,10\right)$. The exact solution is set as follows [21]:

(20) $$\left\{\begin{array}{cc} u_{1} & =-\cos\left(x_{1}\right)\sin\left(x_{2}\right)e^{-2t\nu },\\ u_{2} & =\sin\left(x_{1}\right)\cos\left(x_{2}\right)e^{-2t\nu },\\ p & =-\frac{1}{4}\left[\cos\left(2x_{1}\right)+\cos\left(2x_{2}\right)\right]e^{-4t\nu }. \end{array}\right.$$

The second equation in Equation (19) is the continuity equation for an incompressible fluid and describes the mass conservation of the fluid. From the stream function expression of the two-dimensional incompressible Navier–Stokes equations, one can use a neural network to represent a scalar stream function $\psi (x_{1},x_{2},t)$ and assume $u_{1}=\partial \psi /\partial x_{2}$ and $u_{2}=-\partial \psi /\partial x_{1}$. In this way, we can only consider the residuals of the momentum equation since the continuity of the equation is already fully satisfied.

**Figure 6:**
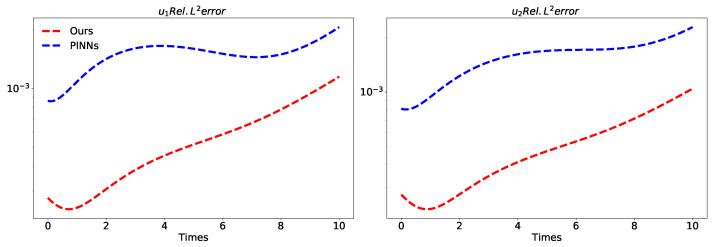
Taylor–Green flow: Rel.$L^{2}$ error over time.

**Figure 7:**
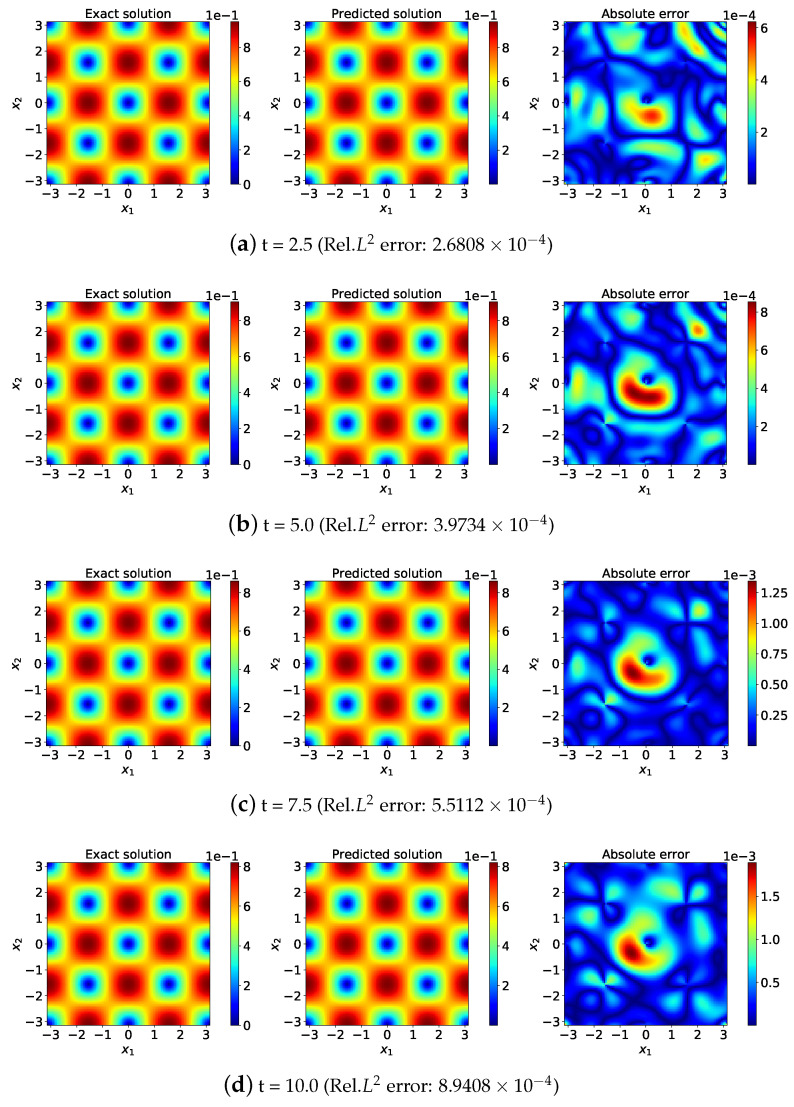
Taylor–Green flow: exact solution, predicted solution, and absolute pointwise error at different times.

In simulations, we set Re=100, and the initial and boundary conditions were obtained using Equation (20). In this example, the swish function is used as the activation function of the neural network, where the value of the scale factor *n* is 20. The training set of the neural network is composed of 10,000 residual training points, 4,000 boundary training points, and 1000 initial training points. We used a neural network with five hidden layers of 50 neurons each to simulate the dynamic behavior described by Equation (19). In Figure 6, we show the Rel.$L^{2}$ error curves of the velocity component compared to those of the PINNs in the left and right panels, respectively. Additionally, the exact solution $U=\sqrt{u_{1}^{2}+u_{2}^{2}}$, the predicted solution $U^{*}=\sqrt{{\left(u_{1}^{*}\right)}^{2}+{\left(u_{2}^{*}\right)}^{2}}$, and the error distributions $|U-U^{*}|$ at four time points are shown in Figure 7. It can be seen that the area with large errors is concentrated in the center, which is caused by the relative error used in the test set in this work, and the value of the denominator at the origin is 0. It can be seen that the proposed method achieves better accuracy than the PINNs in this example.

#### 4.1.3. Cylinder Wake

**Figure 8:**
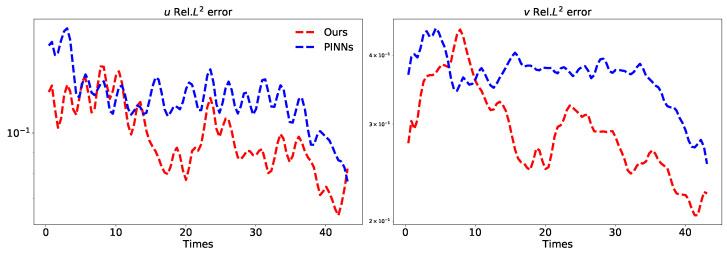
Cylinder wake: Rel.$L^{2}$ error over time.

**Figure 9:**
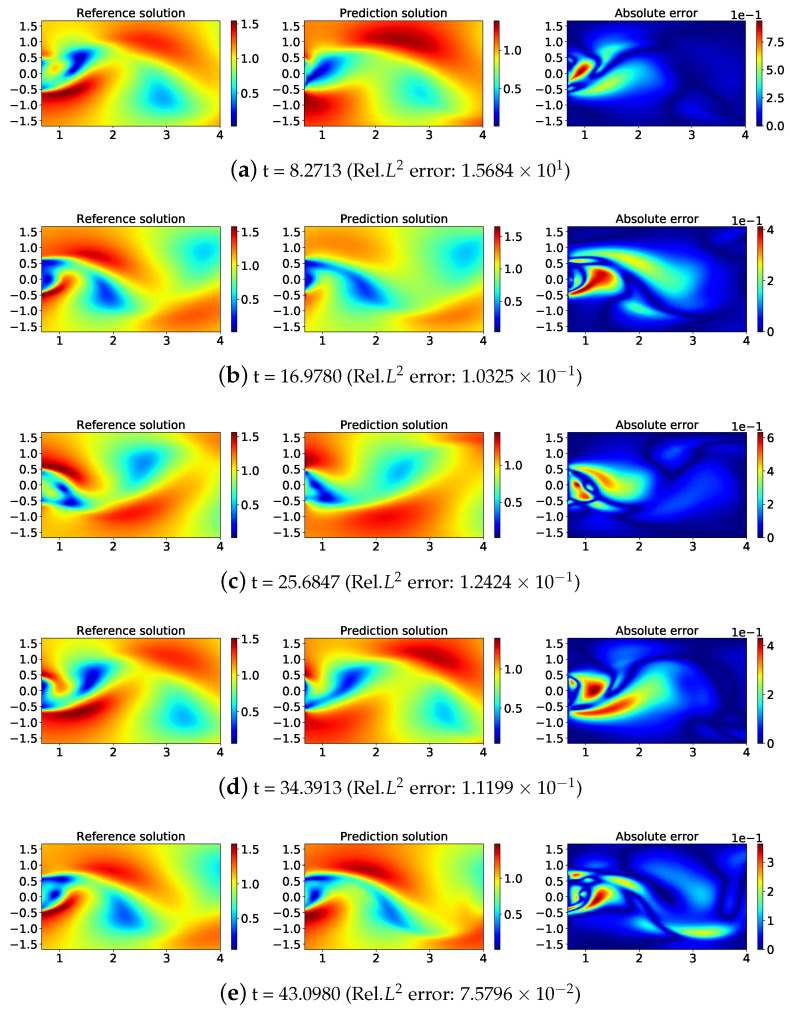
Cylinder wake: reference solution, predicted solution, and absolute pointwise error at different times.

For the third example of the forward problem, we simulated a 2D cylinder wake [63] using the method proposed in this paper. The PDEs’ governing equations are given by Equation (19), and the Reynolds number is Re=3900. High-fidelity data ( the high-fidelity data are from https://github.com/Shengfeng233/PINN-for-turbulence, accessed on 25 September 2022) were used as a reference and provided the boundary and initial data. In this example, the training dataset contains 40,000 residual training points, 10,000 boundary training points, and 5000 initial training points. The structure of the neural network used has 6 hidden layers and 100 neurons in each layer. The tanh function is used as the activation function of the neural network, where the value of the scale factor *n* is 1. In Figure 8, we compare the Rel.$L^{2}$ error of the two velocity components in the left and right graphs, respectively, and in Figure 9, we show the predicted solution obtained by neural network training using the above setup and the corresponding error distribution. It can be seen that the proposed method improves the accuracy by 10%∼30% compared with PINNs. In this example, we found an interesting phenomenon: the error decreases with time on the whole, which seems counterintuitive. The exploration of this problem will help us design better algorithms to further improve the performance of the neural network.

#### 4.1.4. Allen–Cahn Equation

The Allen–Cahn equation is a second-order nonlinear parabolic partial differential equation used to describe the phase separation process in binary alloys and anti-phase boundary movement in crystals [64]. It has been used extensively in different models. We consider an unsteady 3D Allen–Cahn equation as follows:

(21) $$\left\{\begin{array}{cc} u_{t}-\left(u_{x_{1}x_{1}}+u_{x_{2}x_{2}}+u_{x_{3}x_{3}}\right)+u^{3}-u & =f,\left(x_{1},x_{2},x_{3},t\right)\in {\left(-1,1\right)}^{3}\times \left(0,1\right),\\ u & =g,\left(x_{1},x_{2},x_{3},t\right)\in \partial {\left(-1,1\right)}^{3}\times \left(0,1\right),\\ u & =h,\left(x_{1},x_{2},x_{3},t\right)\in {\left(-1,1\right)}^{3}\times \left\{0\right\}. \end{array}\right.$$

**Figure 10:**
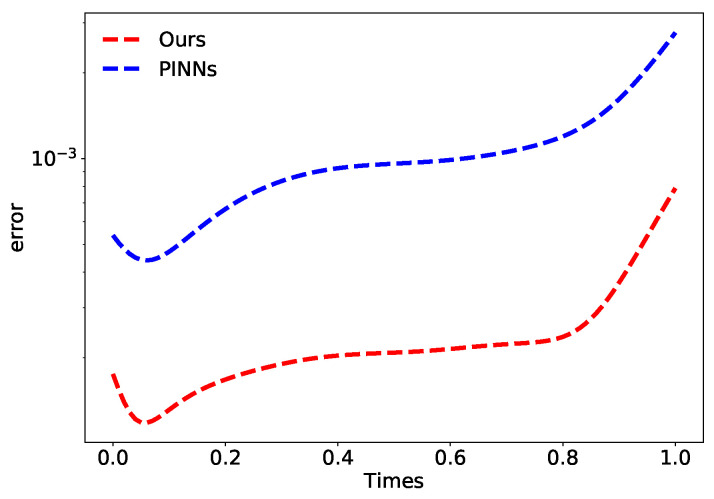
Allen–Cahn equation: Rel.$L^{2}$ error over time.

**Figure 11:**
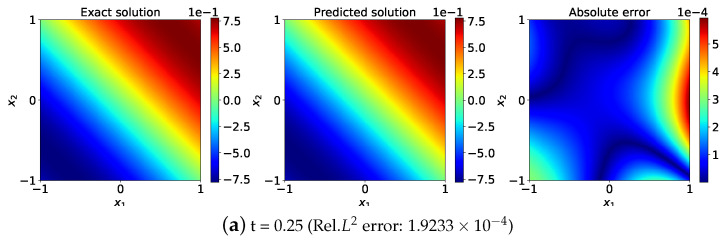

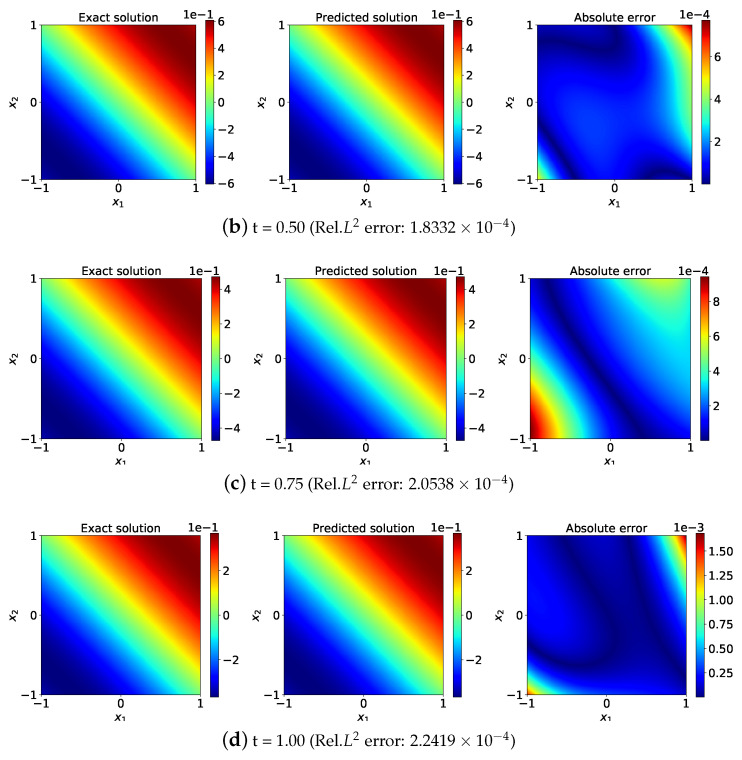
Allen–Cahn equation: exact solution, predicted solution, and absolute pointwise error at different times when $x_{3}=0$.

The source term *f* and the initial boundary value conditions *g* and *h* can be simply obtained from the exact solution: $u\left(x_{1},x_{2},x_{3},t\right)=e^{-t}\sin\left(x_{1}+x_{2}+x_{3}\right)$. We used a neural network with five hidden layers of 50 neurons each. In general, the neural network should have sufficient approximation capacity to consider the predicted complexity of *u*. We obtained the training data points of the neural network by random sampling. In particular, there are 500 initial training points, 2000 boundary training points, and 5000 PDE residual training points in the neural network training set. The value of the hyperparameter $a^{ij}$ is set to 0.1, and the swish function is used as the activation function. The Rel.$L^{2}$ error curves of Equation (21) on the test set are plotted in Figure 10. Compared with PINNs, the accuracy is greatly improved, and the generalization is better. Meanwhile, the exact solution, predicted solution, and error distribution at four time points and $x_{3}=0$ are plotted in Figure 11. Using just a handful of initial data, the method is able to accurately capture the complex nonlinear behavior of the Allen–Cahn equation. Based on the results, it can be seen that the proposed method can accurately produce the solution.

#### 4.1.5. Convection–Diffusion–Reaction Equation

Convection–diffusion–reaction (CDR) equations are basic partial differential equations for the simulation of heat and mass transfer. Their numerical solutions are critical research directions for numerical partial differential equations and computational fluid mechanics, with a broad range of applications [65,66]. For instance, we take the 3D unsteady nonlinear CDR equation as follows:

(22) $$\left\{\begin{array}{cc} u_{t}+\beta \cdot \nabla u-\nu \Delta u+\gamma u^{2} & =f,\left(x_{1},x_{2},x_{3},t\right)\in {\left(-1,1\right)}^{3}\times \left(0,1\right),\\ u & =g,\left(x_{1},x_{2},x_{3},t\right)\in \partial {\left(-1,1\right)}^{3}\times \left(0,1\right),\\ u & =h,\left(x_{1},x_{2},x_{3},t\right)\in {\left(-1,1\right)}^{3}\times \left\{0\right\}, \end{array}\right.$$

where the unknown function *u* usually represents the temperature or concentration of the substance being transferred, $\nu \geq \nu_{0}>0$ is the diffusion coefficient, β is the convective coefficient, and γ is the reaction coefficient. When ν≪max|β|, Equation (22) is called the convection-dominated diffusion problem. In this example, we consider a convection-dominated diffusion problem and use the coefficients $\nu =10^{-4}$, β=(1,1,1), and γ=1, and the exact solution is given as $u\left(x_{1},x_{2},x_{3},t\right)=e^{-t}\arctan\left(x_{1}\right)\arctan\left(x_{2}\right)\arctan\left(x_{3}\right)$. All the right-hand terms can be obtained from the exact solution.

**Figure 12:**
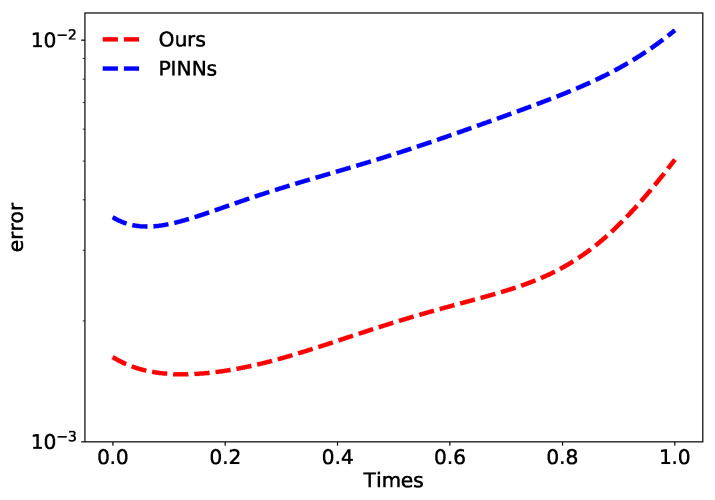
CDR equation: Rel.$L^{2}$ error over time.

**Figure 13:**
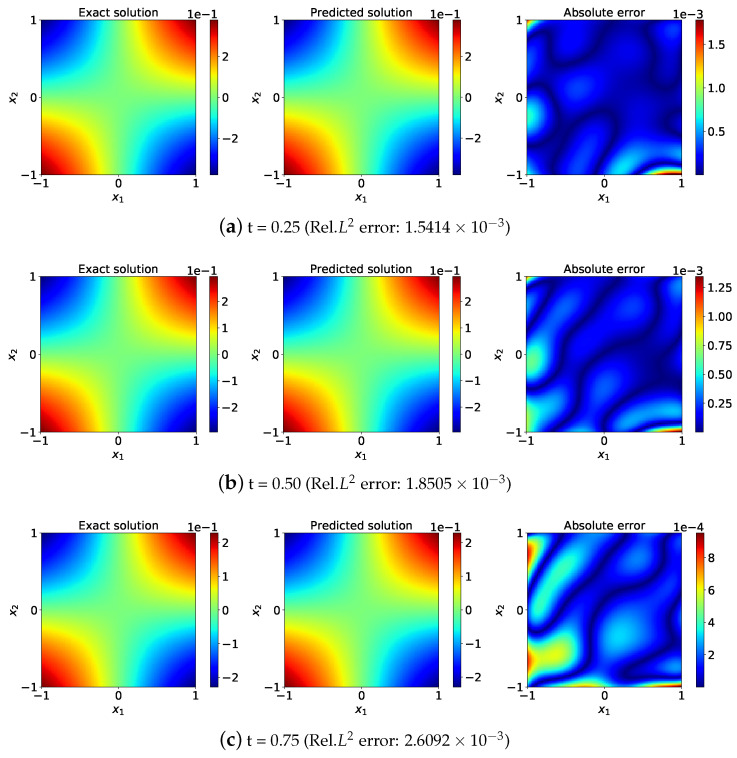

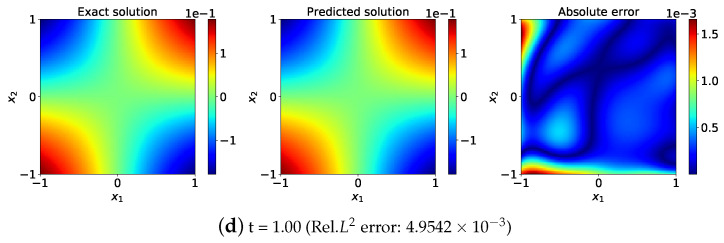
CDR equation: exact solution, predicted solution, and absolute pointwise error at different times when $x_{3}=1$.

We used a training set in which the number of residual points is 5000, 500 data points on ${u|}_{t=0}$ are randomly parsed from the exact solution, and 1000 boundary training points are also given for the boundary conditions of PDEs. We used a four-layer feedforward neural network with 50 neurons in each hidden layer; the value of the scale factor *n* is 100, and the activation function is swish. The Rel.$L^{2}$ error curve on the test set can be seen in Figure 12. Meanwhile, the exact solution, predicted solution, and error distribution at four time points and $x_{3}=1.00$ are plotted in Figure 13. It is clearly observed that solutions with good precision are obtained for this equation.

### 4.2. Inverse Problem

For the inverse problem, the parameter λ in Equation (2) is often unknown. With only a small set of observed data $T_{u}$, the parameter λ can be determined while predicting the entire flow field. To achieve this, we simply add additional terms [23,46] to the loss function (16):

(23) $$L_{u}=\frac{1}{\left|T_{u}\right|}\sum_{\left(x,t\right)\in T_{u}}{\left|u-y\right|}^{2}.$$

#### 4.2.1. Kolmogorov–Petrovskii–Piskunov-Equation

The Kolmogorov–Petrovskii–Piskunov equation (KPP equation) [67] is a kind of nonlinear reaction–diffusion equation commonly used in heat conduction, combustion theory, biology, ecology and other fields. The basic form of the KPP equation is:

(24) $$u_{t}-Du_{x_{1}x_{1}}-au-bu^{m}=0,\;\left(x,t\right)\in \left(-\pi ,\pi \right)\times \left(0,1\right),$$

where *a*, *b*, *D*, and *m* are arbitrary constants, and *m* is not equal to 1. The KPP equation has an exact solution as follows [67]:

$$u\left(x_{1},t\right)={\left[\beta +\exp\left(\lambda t+\frac{\alpha x_{1}}{\sqrt{D}}\right)\right]}^{\frac{2}{1-m}},$$

where the parameters are given by:

$$\begin{array}{cc} \beta & =\sqrt{-\frac{b}{a}},\\ \alpha & =\sqrt{\frac{a{\left(1-m\right)}^{2}}{2(m+1)}},\\ \lambda & =\frac{a(1-m)(m+3)}{2(m+1)}. \end{array}$$

**Figure 14:**
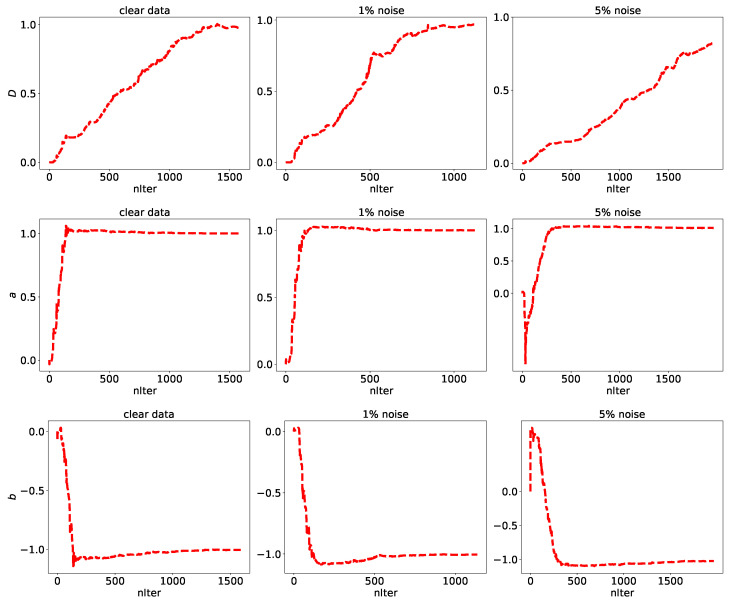
KPP equation: change curves of the parameters *D*, *a*, and *b* with the iteration number.

In this example, we set m=3 and the parameters *D*, *a*, and *b* as unknown parameters in Equation (24). Given scattered and potentially noisy data, our goal is to identify the unknown parameters *D*, *a*, and *b*, as well as to obtain a qualitatively accurate reconstruction of the entire flow field $u(x_{1},t)$. To illustrate the effectiveness of our approach and to highlight the ability of our method to learn from scattered and scarce training data, we created a training dataset by randomly generating $N_{train}=2000$ points across the entire spatiotemporal domain from the exact solution corresponding to D=1, a=1, and b=−1. The neural network architecture used here consists of four hidden layers with 50 neurons in each layer; we set the value of the scale factor *n* to be equal to 50 in Equation (11), and we chose the sigmoid function as the activation function for this example. Upon training, the network was calibrated to predict the entire solution $u(x_{1},t)$, as well as the unknown parameters *D*, *a*, and *b* that define the underlying dynamics. The network can identify the underlying partial differential equations with remarkable accuracy, even when the scattered training data are corrupted by 1% and 5% uncorrelated noise. The results are summarized in Table 1 and in Figure 14, which shows the parameters *D*, *a*, and *b* with the iteration number. It is observed that results with good precision are obtained.

#### 4.2.2. Burgers’ Equation

Then, we take Equation (18) as an example and rewrite it in the following form:

(25) $$u_{t}+\lambda_{1}\left(uu_{x_{1}}+uu_{x_{2}}\right)=\lambda_{2}\left(u_{x_{1}x_{1}}+u_{x_{2}x_{2}}\right),\;\left(x_{1},x_{2},t\right)\in {\left(-1,1\right)}^{2}\times \left(0,1\right).$$

**Figure 15:**
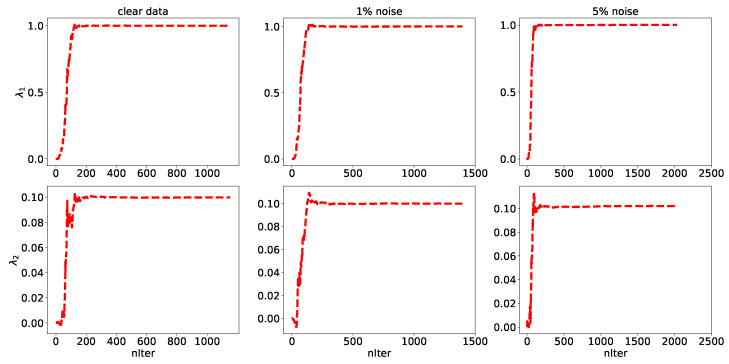
Burgers’ equation: change curves of the parameters $\lambda_{1}$ and $\lambda_{2}$ with the iteration number.

We set the parameters $\lambda_{1}$ and $\lambda_{2}$ as unknown parameters; we still used the same exact solution. To demonstrate how effective the approach is, we created a training dataset by randomly generating $N_{train}=2000$ points on the whole space–time domain from the exact solution with $\lambda_{1}=1$ and $\lambda_{2}=0.1$. We used a neural network with four hidden layers, each with 50 neurons, to predict *u* in the entire computational domain with only a small number of high-fidelity datasets and, at the same time, to determine the unknown parameters $\lambda_{1}$ and $\lambda_{2}$ in Equation (25). The tanh function is set as the activation function, and the value of the scale factor *n* is 20. It is seen in Table 2 that, in this case, the method can correctly identify the unknown parameters $\lambda_{1}$ and $\lambda_{2}$ with very high accuracy, even when the training data are corrupted by noise. The predictions remain robust even when the training data are corrupted by 1% and 5% uncorrelated Gaussian noise. Finally, Figure 15 shows the parameters $\lambda_{1}$ and $\lambda_{2}$ with the iteration number; the results show that the neural network can effectively find the unknown parameters in the equation. The results show that the proposed method makes it possible to obtain great accuracy.

#### 4.2.3. Diffusion–Reaction Equation

**Figure 16:**
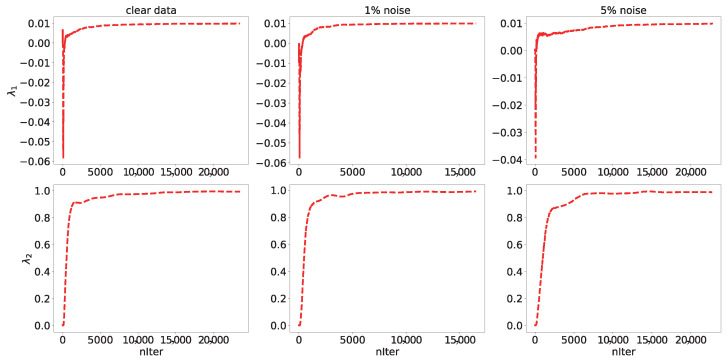
Diffusion–reaction equation: change curves of the parameters $\lambda_{1}$ and $\lambda_{2}$ with the iteration number.

Finally, we consider a 3D unsteady nonlinear diffusion–reaction equation [68] given by:

(26) $$u_{t}-\lambda_{1}\left(u_{x_{1}x_{1}}+u_{x_{2}x_{2}}+u_{x_{3}x_{3}}\right)+\lambda_{2}u^{2}=f,\left(x_{1},x_{2},x_{3},t\right)\in {\left(-1,1\right)}^{3}\times \left(0,1\right),$$

where *u* is the concentration of the substance, and $\lambda_{1}>0$ and $\lambda_{2}>0$ are the diffusion coefficient and reaction coefficient, respectively. The source term *f* can be obtained from the exact solution: $u=e^{-t}\sin\left(\pi x_{1}\right)\sin\left(\pi x_{2}\right)\sin\left(\pi x_{3}\right)$. We randomly selected 5000 training data points from the whole space–time region as the training data for the neural network and constructed a neural network with five hidden layers and 50 neurons in each layer. With only a small amount of data, we simulated the whole flow field and determined the unknown parameters $\lambda_{1}$ and $\lambda_{2}$ in the equation. We used the swish function as the activation function, where the scale factor *n* takes the value 20. Table 3 shows the values of $\lambda_{1}$ and $\lambda_{2}$ ‘discovered’ through neural network training. Additionally, Figure 16 shows the change curves of parameter values in the process of neural network training. It can be seen that neural networks can effectively discover the unknown parameters in Equation (26).

## 5. Conclusions

This paper presents the structure of a second-order neural network constructed through second-order parametric light wave equations. During this work, an adaptive activation function strategy was used to enhance the performance of the neural network, and gPINNs were used to enhance the generalization ability of the neural network. We found that using automatic differentiation techniques to obtain the higher-order derivatives of PDEs’ control equations can cause high computational costs. Therefore, we introduced auxiliary variables to reduce computational costs. Finally, we give some numerical examples. Specifically, for forward problems, we simulated Burgers’ equation, the Allen–Cahn equation, the CDR equation, and the Navier–Stokes equation. Additionally, for inverse problems, our method can effectively determine the unknown parameters of the equation and estimate and reconstruct the unknown parameters with high accuracy.

In this study, we did not consider the balance between the various parts of the loss function in the neural network. The weight setting of different parts has a great impact on the performance of the neural network, but this is beyond the scope of this paper. In the future, we will explore the use of adaptive weight strategy or grid search methods to determine the optimal weight of each term and further study the dependence of neural networks on hyperparameters. The proposed method is combined with other extended PINN algorithms, such as extended physics-informed neural networks (XPINNs), parallel physics-informed neural networks (PPINNs), and so on. In this paper, only the central difference of second-order differential equations is considered for constructing neural networks. We believe that the construction of neural networks using third-order differential equations or higher is also a feasible solution, which will be an upcoming research project. Additionally, we also hope to develop some innovations in theoretical analysis so as to provide a foundation for subsequent research. Similarly, we also found the computational cost caused by using automatic differentiation to calculate derivatives. It will also be useful to study the use of numerical differentiation or Taylor-mode AD to calculate derivatives to prevent the excessive consumption of computing resources.

## Figures and Tables

**Table 1:** KPP equation: Correct values obtained by learning *D*, *a*, and *b* and percentage errors in the identified parameters *D*, *a*, and *b* when the training data are corrupted by different noise levels.

	Clear Data	1% Noise	5% Noise
Exact	(D,a,b) = (1.000,1.000,−1.000)		
Identified	(0.977,1.001,−1.003)	(0.972,1.002,−1.003)	(0.819,1.010,−1.024)
Error	$\left(2.26\times 10^{-2},1.10\times 10^{-3},3.41\times 10^{-3}\right)$	$\left(2.81\times 10^{-2},1.52\times 10^{-3},3.89\times 10^{-3}\right)$	$\left(1.81\times 10^{-1},9.82\times 10^{-3},2.38\times 10^{-2}\right)$

**Table 2:** Burgers’ equation: correct values obtained by learning $\lambda_{1}$ and $\lambda_{2}$ and percentage errors in the identified parameters $\lambda_{1}$ and $\lambda_{2}$ when the training data are corrupted by different noise levels.

	Clear Data	1% Noise	5% Noise
Exact	$\left(\lambda_{1},\lambda_{2}\right)$ = (1.000,0.100)		
Identified	(1.0001,0.0999)	(0.9991,0.1001)	(1.0020,0.1022)
Error	$\left(1.81\times 10^{-4},3.21\times 10^{-4}\right)$	$\left(5.95\times 10^{-4},7.39\times 10^{-3}\right)$	$\left(2.02\times 10^{-3},2.16\times 10^{-2}\right)$

**Table 3:** Diffusion–reaction equation: correct values obtained by learning $\lambda_{1}$ and $\lambda_{2}$ and percentage errors in the identified parameters $\lambda_{1}$ and $\lambda_{2}$ when the training data are corrupted by different noise levels.

	Clear Data	1% Noise	5% Noise
Exact	$\left(\lambda_{1},\lambda_{2}\right)$ = (0.0100,1.0000)		
Identified	(0.0099,0.9924)	(0.0098,0.9914)	(0.0099,0.9878)
Error	$\left(1.31\times 10^{-2},7.60\times 10^{-3}\right)$	$\left(1.53\times 10^{-2},8.58\times 10^{-3}\right)$	$\left(1.36\times 10^{-2},1.22\times 10^{-2}\right)$

## Data Availability

The data that support the finding of this study are available from the corresponding author upon reasonable request.

## Appendix A. Computational Cost Calculation

In this part, we use Burgers’ Equation (18) as an example to calculate the computational cost of the method used. We used a neural network with only three hidden layers, each with only 50 neurons. The training dataset used included 500 initial training data points, 1000 boundary training points, and 5000 residual training points for the PDEs’ governing equations. We used the Adam algorithm to train 50 k times. The training results are shown in Table A1. We compared the reference PINN equation, the adaptive PINN equation based on Neuron-wise locally adaptive activation functions (N-LAAF-PINNs), gPINNs, and the methods mentioned in Section 3. It can be seen from the results that both the adaptive activation function strategy and the gradient enhancement strategy can improve the prediction accuracy of the neural network, but the gradient enhancement strategy is time-consuming. We also performed the test without the MIM strategy, which doubled the prediction accuracy but also doubled the training time. This is only a preliminary comparison. As part of future work, we will summarize the existing algorithms and provide a large number of numerical examples for comparative analysis. Although this is a time-consuming process, it is also very important. This research content will help us to design appropriate algorithms for specific problems.

**Table A1:** Burgers’ equation: Computational accuracy and computational cost calculations.

t	2.0	4.0	6.0	8.0	10.0	Training Times
PINNs	$1.48\times 10^{-3}$	$1.32\times 10^{-3}$	$1.26\times 10^{-3}$	$1.31\times 10^{-3}$	$1.52\times 10^{-3}$	456.46
gPINNs	$4.26\times 10^{-4}$	$4.84\times 10^{-4}$	$4.26\times 10^{-4}$	$3.49\times 10^{-4}$	$3.85\times 10^{-4}$	3042.68
N-LAAF-PINNs	$1.78\times 10^{-4}$	$1.56\times 10^{-4}$	$1.41\times 10^{-4}$	$1.34\times 10^{-4}$	$1.41\times 10^{-4}$	1228.73
Ours	$7.52\times 10^{-5}$	$7.16\times 10^{-5}$	$7.43\times 10^{-5}$	$8.26\times 10^{-5}$	$9.64\times 10^{-5}$	1804.28
Ours (Without MIM)	$3.28\times 10^{-5}$	$3.58\times 10^{-5}$	$3.74\times 10^{-5}$	$5.00\times 10^{-5}$	$6.94\times 10^{-5}$	3918.47
